# Dyadic associations between marital satisfaction and coparenting quality: gender differences and the moderating role of caregiving identity

**DOI:** 10.3389/fsoc.2024.1422404

**Published:** 2024-08-01

**Authors:** Patty X. Kuo, Weiman Xu, Zhenqiao Yang

**Affiliations:** Department of Child, Youth and Family Studies, University of Nebraska-Lincoln, Lincoln, NE, United States

**Keywords:** coparenting, marital satisfaction, mothers, fathers, caregiving identity

## Abstract

Our study investigated the contribution of caregiving identity in the association between marital satisfaction and coparenting quality in fathers and mothers from a sample of opposite-sex couples of young children living in different areas of the United States. We conducted nested Actor-Partner Interdependence Models and moderation tests to examine potential differences between fathers and mothers in associations between marital satisfaction and coparenting quality, as well as the role of caregiving identity in the association. Results confirmed gender differences in the association between marital satisfaction and coparenting. Both mother’s and father’s caregiving identity interacted with their own marital satisfaction, but these interactions only impacted the coparenting quality reported by mothers. Additionally, caregiving identity in fathers and mothers was associated with the coparenting quality reported by their spouses. Our study highlighted the important role of caregiving identity in understanding the relation between marital satisfaction and coparenting quality in the intrafamilial processes of couples with young children.

## Introduction

The degree to which individuals can effectively coparent together primarily depends on the couple relationship, according to the Ecological Model of Coparenting (Feinberg, 2003). Coparenting quality describes how parents support each other in their role as parents and is a component, but distinct from the overall couple relationship (Feinberg, 2003). However, marital satisfaction (i.e., satisfaction with the marital couple relationship) impacts coparenting quality (Feinberg, 2003). Recent meta-analytic evidence revealed that parent gender moderates the association between marital satisfaction and coparenting quality (Ronaghan et al., 2024), with slightly larger associations for mothers (*r* = 0.48) compared to fathers (*r* = 0.42), revealing potentially different couple-coparenting processes for women and men, and these gender differences are not articulated in predominating theory about coparenting processes. These results also call into question some predominating frameworks for understanding fathers as coparents. According to the Father Vulnerability hypothesis, fathers’ coparenting is supposedly more vulnerable to marital discord than mothers’ because of their relatively weaker socialization into caregiving and fathering compared to women’s lifetime socialization into mothering and caregiving roles (Cummings and Davies, 2010). A critical argument in this hypothesis is that a strong caregiving identity (i.e., importance of engaging in caregiving in their role as a parent, Maurer et al., 2001) buffers their parenting from problems in the couple relationship, and the reason for father vulnerability is because men have not been adequately socialized into caregiving roles (Cummings and Davies, 2010).

Maternal gatekeeping, or the deliberate exclusion of fathers from caregiving, offers another explanation for gendered differences in couple-coparenting processes, particularly among women who view caregiving as critical to their own parenting identities (Allen and Hawkins, 1999). However, cultural movement toward involved fathering in the U.S. and more gender egalitarian countries in Europe have included heightened expectations for more overlap between mothers and fathers’ responsibilities as parents (Fagan et al., 2014; Schoppe-Sullivan and Fagan, 2020; Volling and Palkovitz, 2021). These expectations include fathers actively engaging in caregiving for their children (e.g., feeding, dressing, and coordinating children’s schedules). Fathers as active coparents is part of a gender role shift in families (Volling and Palkovitz, 2021; Campbell, 2023). While egalitarian gender role beliefs appear to improve coparenting quality (Kuo et al., 2017; Campbell, 2023), we surmise that these effects are not driven by all facets of gender role beliefs, which encompass perceived appropriate conduct in multiple domains such as sex, emotionality, and typical activities based on gender. Indeed, the concept of caring masculinities also allows for simultaneous inclusion of traditional gender role beliefs such as men’s responsibility for protection and provision along with centering caregiving (Elliott, 2016). Thus, if caregiving is no longer specifically tied to gender roles for women and men, then the relative impact of caregiving identity on coparenting should be consistent across fathers and mothers. Whereas previous research that has found gender differences in couple-coparenting processes and hypothesized differences in mothers’ and fathers’ parenting identities as a potential mechanism for gender differences (Le et al., 2016; Peltz et al., 2018), we are directly testing the proposed underlying processes that contribute to gender differences within father vulnerability (fathers have weak caregiving identities) and maternal gatekeeping (mothers have strong caregiving identities) by incorporating caregiving identity as a moderator. Our primary aim in the present study was to investigate the unique contributions of marital satisfaction, parents’ caregiving identity, and the interaction between marital satisfaction and caregiving identity to coparenting quality in the parent dyad. Our secondary aim was to evaluate gender differences. Aligning with recent meta-analytic evidence (Ronaghan et al., 2024), we hypothesized marital satisfaction would predict coparenting quality, with stronger effect sizes for mothers compared to fathers. We also hypothesized that stronger caregiving identity would be related to better coparenting quality, regardless of parents’ gender. Finally, we hypothesized that caregiving identity would mitigate associations between marital satisfaction and coparenting quality.

## Materials and methods

Data came from a multi-phase online study which was designed to study parenting stress in couples of young children (Kuo and Johnson, 2021; Johnson et al., 2023; Kuo et al., 2023) and received ethical approval from University of Notre Dame’s Institutional Review Board. The criteria of eligibility for participation included that parents were living in the U.S., aged 18 years or older, cohabitating with opposite-sex partners, and parenting at least one child aged 6 years or younger. Potential participants and their spouses needed to be enrolled together. Each parent was expected to complete all measures independently from their partners. The study included baseline surveys and subsequent daily diaries on mood, stressors, and familial emotional climate. A rigorous screening was conducted to prevent fraudulent and bot responses. Interested parents were first required to fill out a contact information form on a separate website from the Qualtrics survey. After the consent, participants were asked for provision of contact to their partner or spouse, who were reached directly by the first author and asked to complete the same screening questionnaire and consent form. Matching information was required for each pair of parents to proceed to enrollment. Each participant was compensated with a $5 gift card for completing the 20-min baseline survey, a $1 gift card per 5-min diary survey up to 10 days, and eligibility to a drawing for a $100 gift card for couples with full survey completion. The current study analysis included baseline data only. Two hundred and two parents (101 couples) were enrolled in the project, and 198 parents (99 couples) completed the full baseline survey.

Most participants (89.7%) were married, and the couples averaged 9.89 (*SD* = 4.87) years in relationship. The children in the study included 114 boys and 110 girls, who were 3.22 (*SD* = 2.33) years old on average. Couples had one to seven (*M* = 2.24, *SD* = 1.31) children in the family. Most mothers (98.0%) and all fathers lived with at least one biological child. Both mothers and fathers were highly educated with 76.0% of mothers and 70.4% of fathers holding at least a bachelor’s degree. There was a high racial composition of White parents (87.1% of mothers; 89.1% of fathers), followed by Black/African-American (seven mothers; three fathers), Asian (three mothers; four fathers), and others racial group (two mothers; one father). Five mothers and two fathers identified themselves as Hispanic. Fathers and mothers differed in working status and role status. There were 84.7% of fathers but 36.4% of mothers working full-time, more mothers (15.2%) worked part time than fathers (4.1%), and 43.4% of mothers but only 3.1% of fathers reported to be “homemakers.” Household income ranged from $20,000 to $120,000 and up, with the median income range of $70,000– $79,000. Participants were living in all areas of the U.S., including the Midwest (65.0%), the South (16.0%), the Northeast (13.0%), and the West (6.0%).

*Coparenting quality* was assessed with the Parental Alliance Measure (Abidin and Brunner, 1995; Abidin and Konold, 1999). This measure includes 20 items to assess parents’ perception of teamwork with parenting partners. Each item (e.g., “*My child’s other parent believes I am a good parent*”) was responded to on a 5-point Likert scale (1 = *strongly disagree* to 5 = *strongly agree*), regarding responders’ agreement with the item statement. A mean score was calculated for each individual based on 20 items, with higher scores indicating higher levels of coparenting quality. The internal consistency of the measure was good for mothers (*ɑ* = 0.95) and fathers (*ɑ* = 0.92).

*Marital* sa*tisfaction* was measured by the well-validated Kansas Marital Satisfaction Scale (Schumm et al., 1986). This measure consists of three items on people’s satisfaction with spouses, marriage, and marital relationship. Participants responded to each item (e.g., *How satisfied are you with your marriage?*”) using a 7-point Likert scale (1 = *not at all* to 7 = *extremely*). Item scores were averaged to indicate participants’ marital satisfaction levels, with higher scores represent higher satisfaction levels. The measure exhibited good internal consistency in the current study (mothers’ *ɑ* = 0.97 and fathers’ *ɑ* = 0.95).

*Caregiving identity* was assessed by the Caregiving Identity subscale of the Caregiving and Breadwinning Identity and Reflected-Appraisal Inventory (Maurer et al., 2001). This subscale included 14 items asking about parents’ commitment as a child caregiver. Parents rated their agreement with each item, (e.g., *I should be committed to actively meeting my child’s physical needs*) from 1 = *strongly disagree* to 5 = *strongly agree*. Mean scores of the 14 items were used to indicate caregiving identity levels (mothers’ *ɑ* = 0.65 and fathers’ *ɑ* = 0.73), with a higher score representing a stronger caregiving identity. Previous studies reported the Cronbach’s alpha of the subscale to be 0.74 in fathers (Nguyen, 2018) and similar values in combined samples of mothers and fathers [0.75 in Maurer et al. (2001) and 0.74 in Maurer and Pleck (2006)].

## Results

### Preliminary analyses

See Table 1 for descriptive statistics and correlations of all main study variables. We examined potential covariates among several parent and child demographic variables for mother and father variables on coparenting quality. Pearson’s correlation tests were conducted for continuous, potential covariates, including parents’ age, education, family income, years of cohabitation, and number of children in the family. None of these were significantly correlated with the outcome variables (i.e., father coparenting or mother coparenting; *p*s ranged from 0.18 to 0.85). ANOVA was conducted for categorical variables including ethnicity and residential region. Results indicated non-significant differences in coparenting across ethnicity (*p*s ranged from 0.53 to 0.80) or residential region (*p*s ranged from 0.58 to 0.76). *T*-tests were used for binary variables, mothers’ and fathers’ work status (full time vs. not full time), and no significant results were found (*p*s ranged from 0.30 to 0.71). Overall, none of the potential covariates significantly related to coparenting quality in our sample. In addition, we examined the missing value patterns of our data and conducted the Little’s missing completely at random (MCAR) tests (Little, 1988; Koptur, 2022). Results suggested that our data is MCAR. Therefore, none of these potential covariates or missingness were controlled for in the following analyses.

**Table 1:** Descriptive statistics and correlations among the study variables.

	1	2	3	4	5	6
1. Mother’s marital satisfaction						
2. Father’s marital satisfaction	0.35^***^					
3. Mother’s caregiving identity	0.09	−0.03				
4. Father’s caregiving identity	0.15	−0.002	−0.35^***^			
5. Mother–reported coparenting quality	0.61^***^	0.37^***^	−0.08	0.32^***^		
6. Father–reported coparenting quality	0.35^***^	0.40^***^	0.10	0.18	0.57^***^	
*M*	6.06	6.12	4.08	3.57	4.37	4.32
*SD*	1.14	1.15	0.38	0.42	0.57	0.48

^***^p < 0.001.

Using *Mplus* 8.8 Muthén and Muthén (1998-2022), we conducted a pair of nested path models to test the standard equal variance assumption in Actor-Partner Interdependence Models (APIM; Gonzalez and Griffin, 2012) for our APIM Moderation Model (APIMoM; Garcia et al., 2015). Specifically, variances were constrained to be equal of each independent and dependent variable across spouses, and then released for free estimation to test this assumption. Considering the nonnormality of some study variables, we used maximum likelihood estimation with robust standard errors (MLR in *Mplus*). The Chi-Square Difference test (Satorra and Bentler, 2010; Bryant and Satorra, 2012) was calculated using formulas suggested by Asparouhov and Muthén (2010). The difference test was non-significant, meaning that our model met the equal variance assumption in APIMs and that the equality constraints on the variances should be retained for hypothesis testing (Gonzalez and Griffin, 2012).

### Hypothesis testing

Our study aims were to (1) investigate the unique contributions of marital satisfaction and parents’ caregiving identity to coparenting quality, and the moderating role of caregiving identity on associations between marital satisfaction and coparenting quality; and (2) to evaluate gender differences in these processes. Testing for potential gender differences requires conducting a series of nested APIM models and statistically comparing models that impose equality constraints on paths between mothers and fathers (hypothesis: gender equivalence), and a model that does not have equality constraints between mothers and fathers (hypothesis: gender difference). Our base model was the total gender difference model. It included (1) actor and partner paths from marital satisfaction and caregiving identity to coparenting quality and (2) interaction terms between each parent’s own marital satisfaction and their own caregiving identity on their reported coparenting quality and their partner’s reported coparenting quality. There were no equality constraints imposed and our base model showed excellent fit, *χ*^2^(11) = 9.08, *p* = 0.62, RMSEA = 0.00, CFI = 1.00.

The marital satisfaction and caregiving identity gender equivalence model was used to test the alternative hypothesis that there were no gender differences in caregiving identity-coparenting paths and marital satisfaction-coparenting paths for both mothers and fathers. Equality constraints were placed on each of the marital and the caregiving identity paths predicting coparenting quality (i.e., mother actor path = father actor path; mother partner path = father partner path). No equality constraints were placed on the interaction paths. We compared the marital satisfaction and caregiving identity gender equivalence model [*χ*^2^(15) = 20.47, *p* = 0.15, RMSEA = 0.06, CFI = 0.95] with the total gender difference model. The Satorra-Bentler Scaled Chi-Square difference test revealed significant differences in model fit [Δ*χ*^2^(4) = 21.39, *p* < 0.001], meaning that the total gender difference model fit the data better than the marital satisfaction and caregiving identity gender equivalence model.

To attempt to isolate the patterns of gender differences, we then compared our total gender difference model to a model that tested gender equivalences in the patterns of associations for marital satisfaction and coparenting by releasing equality constraints on the caregiving identity paths but keeping the constraints on the marital satisfaction paths. The total gender difference model fit better than the gender equivalence in marital satisfaction model [Δ*χ*^2^(2) = 41.01, *p* < 0.001], evincing that gender differences existed for associations between marital satisfaction and coparenting. However, results suggested that there was no statistically significant difference between the total gender difference model and the gender equivalence in caregiving identity model [Δ*χ*^2^(2) = 3.70, *p* = 0.16]. This means that there are no gender differences in the caregiving identity-coparenting paths between mothers and fathers.

In summary, our nested model comparisons indicated that while there were gendered patterns for marital satisfaction and coparenting, the effect sizes predicting coparenting from caregiving identity were statistically nonsignificant between mothers and fathers. Recommended procedures for model selection for results interpretation among nested models is to choose the more parsimonious model if there is no significant chi-square difference in model fit between models (Gonzalez and Griffin, 2012). As a result, we chose the caregiving identity gender equivalence model for final interpretation.

**Figure 1:**
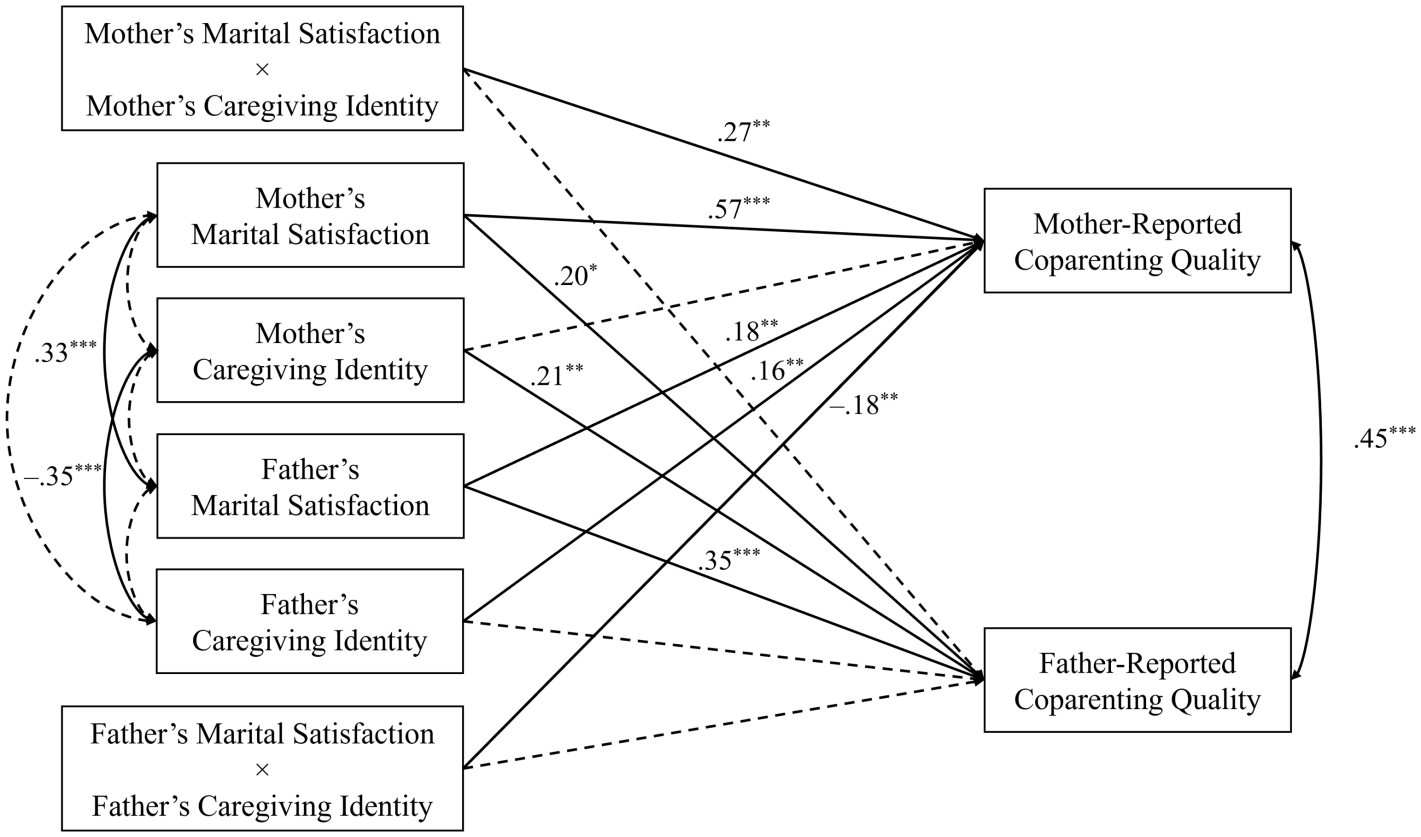
Standardized coefficients in final APIMoM (*N* = 94). ^*^p < 0.05, ^**^p < 0.01, ^***^p < 0.001. Statistically significant paths are shown in solid lines and non-significant paths are shown in dashed lines.

**Figure 2:**
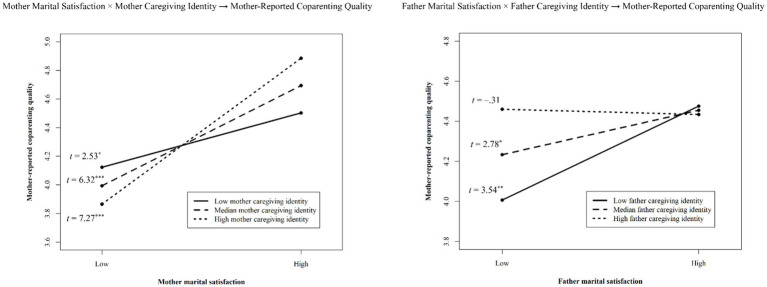
Interaction effects. ^*^p < 0.05, ^**^p < 0.01, ^***^p < 0.001.

Table 2, Figure 1 shows the estimates for our final model. In this model, caregiving identity exerted significant partner effects (e.g., fathers’ caregiving identity significantly predicted mother-reported coparenting quality), but no significant actor effects. Marital satisfaction exerted significant actor (e.g., mother’s marital satisfaction predicting her own reports of coparenting quality) and partner paths. Mothers’ reported coparenting quality was also significantly predicted by two interactions that affected the coparenting quality reported by mothers: one between mother’s marital satisfaction and caregiving identity; the other between father’s marital satisfaction and caregiving identity. *Post hoc* simple slopes tests of these interactions revealed significant, positive slopes for mothers’ caregiving identity and marital satisfaction (See Figure 2). Across all levels of mothers’ caregiving identity, as marital satisfaction increased, mothers’ reported coparenting quality also increased, but the slopes were steeper for mothers with higher caregiving identities. The same was not true for fathers’ caregiving identity. Mothers partnered with fathers who had low or median caregiving identity reported higher coparenting quality as his marital satisfaction increased. Mothers partnered with high caregiving identity fathers reported greater coparenting quality, regardless of fathers’ own marital satisfaction. There were no significant interactions predicting fathers’ coparenting quality.

**Table 2:** Coefficients in the final APIMoM, with imposed equality constraints on caregiving identity paths only (*N* = 94).

Regression coefficients	*b*	*S.E.*	95% CI	*β*
*Mother-reported coparenting quality*				
Intercept	4.34^***^	0.04	[4.27, 4.42]	7.11
Actor paths				
Mother marital satisfaction	0.31^***^	0.05	[0.21, 0.40]	0.57
Mother caregiving identity	0.08	0.08	[−0.08, 0.24]	0.05
Mother marital satisfaction × mother caregiving identity	0.36^**^	0.11	[0.15, 0.57]	0.27
Partner paths				
Father marital satisfaction	0.10^**^	0.04	[0.03, 0.17]	0.18
Father caregiving identity	0.25^**^	0.08	[0.09, 0.41]	0.16
Father marital satisfaction × father caregiving identity	−0.26^**^	0.08	[−0.42, −0.10]	−0.18
*Father-reported coparenting quality*				
Intercept	4.33^***^	0.04	[4.24, 4.41]	9.25
Actor paths				
Father marital satisfaction	0.15^***^	0.04	[0.07, 0.22]	0.35
Father caregiving identity	0.08	0.08	[−0.08, 0.24]	0.07
Father marital satisfaction × father caregiving identity	−0.17	0.10	[−0.37, 0.04]	−0.15
Partner paths				
Mother marital satisfaction	0.08^*^	0.04	[0.01, 0.16]	0.20
Mother caregiving identity	0.25^**^	0.08	[0.09, 0.41]	0.21
Mother marital satisfaction × mother caregiving identity	0.02	0.10	[−0.19, 0.22]	0.02

^*^p < 0.05, ^**^p < 0.01, ^***^p < 0.001.

## Discussion

Our results about marital satisfaction and coparenting quality mirrored findings from a recent meta-analysis that showed positive associations between marital satisfaction and coparenting quality (Ronaghan et al., 2024). Here, we found the expected positive associations in both actor (one person’s marital satisfaction predicting their own reported coparenting quality) and partner effects (one person’s marital satisfaction predicting their spouse’s reported coparenting quality). Whereas the meta-analysis used to compare gender differences in samples including only mothers and only fathers showed that there were larger effects of marital satisfaction on coparenting quality for mothers than fathers (Ronaghan et al., 2024), our study is showing significant gender differences in these associations even *within* families. Thus, there are likely some gendered processes that are occurring in the marital and coparenting subsystems – but not that of father vulnerability, which was previously proposed (Cummings and Davies, 2010). Instead, mothers’ reported coparenting quality appeared to be more strongly related to marital satisfaction than for fathers.

Findings from the study suggested that caregiving identity impacted mothers’ perceptions of the coparenting relationship in more nuanced ways than for fathers. Although mother’s and father’s reported coparenting quality were both predicted by spouse caregiving identity, mother-reported coparenting quality was also affected by interactions of caregiving identity and marital satisfaction in themselves and their partners. However, these interaction effects were not significant for fathers’ reported coparenting quality. Park et al. (2010) argued that despite trends toward increasingly egalitarian division of labor between women and men in parenting roles, stereotyped experiences persisted regarding parental identity and parenting experiences, and there continued to be stronger implicit effects of parental identities in women than men (Park et al., 2010; Hodges and Park, 2013). Our findings seemed to align with this argument that mothers were more susceptible to the influences of their own and their partners’ caregiving identity.

Several researchers have also proposed that the gendered process arises from differences in caregiving responsibilities and the relative importance of caregiving identities for mothers, compared to fathers (Cummings and Davies, 2010; Le et al., 2016; Ronaghan et al., 2024). Contrasting theorists claimed that as fathers and mothers’ roles become more similar over time (Fagan et al., 2014), gendered differences seen in studying parenting may cease to exist. Our paper’s key novel contribution was to examine the role of caregiving identity on coparenting quality and whether caregiving identity could moderate associations between marital satisfaction and coparenting quality. Here, we found that both mothers and fathers reported a higher quality coparenting relationship when their *partners* held stronger caregiving identities. This means that when a parent feels a personal responsibility toward caregiving, the other parent is likely to see their partners as a supportive coparent, regardless of gender (Figure 1, Table 2).

Caregiving identity also moderated associations between marital satisfaction and coparenting quality, but for mothers’ reported coparenting quality only. Although all mothers reported lower quality coparenting relationships when they were also in unsatisfactory marriages, our cross-over interaction results revealed that the marital and coparenting subsystems appeared to be more tightly connected for mothers with stronger caregiving identities. This link was attenuated (i.e., slope was flatter) among mothers with lower caregiving identities. Previous work has also found longitudinal associations from coparenting at a previous point predicting mothers’ marital satisfaction, but not fathers, hinting that coparenting quality may actually be driving mothers’ marital satisfaction (Le et al., 2016; Peltz et al., 2018), rather than the reverse. Predominating theories on the ecology of coparenting do not assume gender differences (Feinberg, 2003), but evolutionary biosocial theories do. According to parental investment theory (Trivers, 1972), due to biologically-based differences in reproduction between males and females, women have evolved to select men that would be more invested in resource provision and care. In modern day terms, this means a better coparent. If we assume that coparenting quality is the basis of marital satisfaction for women, women with stronger caregiving identities may need additional support from their spouses in their role as a parent to feel satisfied with their marriages overall.

Father’s caregiving identity also moderated the partner effect of father’s marital satisfaction on mother’s reported coparenting quality. We found fathers’ marital satisfaction was no longer associated with mothers’ reported coparenting quality (i.e., nonsignificant slope) when fathers reported having a high caregiving identity. These results show that fathers’ caregiving identity can buffer potential negative impacts of an unsatisfactory marriage on the coparenting relationship. We contend that fathers with stronger caregiving identities are more likely to actively be supportive coparents.

Our study has several limitations to consider, including constraints on generality, based on sample characteristics (e.g., all parents in opposite-sex relationships; majority white, highly educated). We also note that a slightly substandard alpha for the caregiving identity measure for mothers. Using a significance level of 0.05 and to achieve a power level of 0.80 for analyses, a sample size of 108 is required (Cohen, 1988). However, our current sample size is 94 participants, which might lead to type II error.

While our findings shed light on gendered processes in coparenting quality, if parental roles between mothers and fathers are becoming more similar in some families, we wonder why there are still overall gender differences in the magnitude of associations between marital satisfaction and coparenting. Perhaps a strong identification with gendered roles (i.e., mothers as primary caregivers) leads women to place greater importance on parenting and coparenting as factors contributing to marital satisfaction. As trends toward intensive parenting increase (Cha and Park, 2021), we contend that both mothers’ and fathers’ caregiving identities will become more critical to marital and coparenting family processes. Family professionals can invite couples to engage in conversations about their own roles as parents, and what each person needs from their partner to feel supported in their parental role as a way to strengthen the overall couple relationship.

## Data availability statement

The raw data supporting the conclusions of this article will be made available by the authors, without undue reservation.

## Ethics statement

The studies involving humans were approved by the University of Notre Dame Institutional Review Board. The study were conducted in accordance with the local legislation and institutional requirements. The participants provided their written informed consent to participate in this study.

## Author contributions

PK: Conceptualization, Data curation, Formal analysis, Funding acquisition, Methodology, Project administration, Supervision, Writing – original draft, Writing – review & editing. WX: Formal analysis, Writing – original draft, Writing – review & editing. ZY: Formal analysis, Writing – original draft, Writing – review & editing.

## References

[ref1] Abidin R. R. Brunner J. F. (1995). Development of a parenting alliance inventory. J. Clin. Child Psychol. 24, 31–40. doi: 10.1207/s15374424jccp2401_4

[ref2] Abidin R. R. Konold T. R. (1999). Parenting alliance measure: Professional manual. Florida: Psychological Assessment Resources. Inc.

[ref3] Allen S. M. Hawkins A. J. (1999). Maternal gatekeeping: mothers’ beliefs and behaviors that inhibit greater father involvement in family work. J. Marriage Fam. 61, 199–212. doi: 10.2307/353894

[ref4] Asparouhov T. Muthén B. (2010). Computing the strictly positive Satorra-Bentler chi-square test in Mplus. Mplus Web Notes 12, 1–12.

[ref5] Bryant F. B. Satorra A. (2012). Principles and practice of scaled difference Chi-Square testing. Struct. Equ. Model. Multidiscip. J. 19, 372–398. doi: 10.1080/10705511.2012.687671

[ref6] Campbell C. G. (2023). Two decades of coparenting research: a scoping review: Marriage & Family Review. Marriage Fam. Rev. 59, 379–411. doi: 10.1080/01494929.2022.2152520

[ref7] Cha Y. Park H. (2021). Converging educational differences in parents time use in developmental child care. J. Marriage Fam. 83, 769–785. doi: 10.1111/jomf.12720

[ref9003] Cohen J. (1988). Statistical power analysis for the behavioral sciences (2nd ed.). Hillside, NJ: Lawrence Erlbaum Associates.

[ref8] Cummings E. M. Davies P. T. (2010). Marital conflict and children: An emotional security perspective. New York, NY: Guilford Press.

[ref9] Elliott K. (2016). Caring masculinities: theorizing an emerging concept. Men Masculinities 19, 240–259. doi: 10.1177/1097184X15576203

[ref10] Fagan J. Day R. Lamb M. E. Cabrera N. J. (2014). Should researchers conceptualize differently the dimensions of parenting for fathers and mothers? J. Fam. Theory Rev. 6, 390–405. doi: 10.1111/jftr.12044

[ref11] Feinberg M. E. (2003). The internal structure and ecological context of Coparenting: a framework for research and intervention. Parenting 3, 95–131. doi: 10.1207/S15327922PAR0302_01, PMID: 21980259 PMC3185375

[ref12] Garcia R. L. Kenny D. A. Ledermann T. (2015). Moderation in the actor–partner interdependence model. Pers. Relat. 22, 8–29. doi: 10.1111/pere.12060

[ref13] Gonzalez R. Griffin D. (2012). “Dyadic data analysis” in APA handbook of research methods in psychology, Vol 3: Data analysis and research publication Eds. Cooper, H., Camic, P. M., Long, D. L., Panter, A. T., Rindskopf, D., and Sher, K. J. (Washington, DC: American Psychological Association), 439–450.

[ref14] Hodges A. J. Park B. (2013). Oppositional identities: dissimilarities in how women and men experience parent versus professional roles. J. Pers. Soc. Psychol. 105, 193–216. doi: 10.1037/a0032681, PMID: 23713699 PMC3722311

[ref15] Johnson V. J. Choi D. Wheeler L. A. Kuo P. X. (2023). Coparenting support in the context of difficult children: mother and father differences. Fam. Process:e12911. doi: 10.1111/famp.12911, PMID: 37400272

[ref16] Koptur M. (2022) *Murat Koptur Data Science Blog & Projects - Don’t impute all missing data: Missing Data Patterns*. Available at: https://muratkoptur.com/MyDsProjects/MissingData/Analysis.html (Accessed July 1, 2024).

[ref17] Kuo P. X. Johnson V. J. (2021). Whose parenting stress is more vulnerable to marital dissatisfaction? A within-couple approach examining gender, cognitive reappraisal, and parental identity - Kuo - 2021 - family process - Wiley online library. Fam. Process 60, 1470–1487. doi: 10.1111/famp.12642, PMID: 33704779

[ref18] Kuo P. X. Lee K. Johnson V. J. Starr E. J. (2023). Investigating moderators of daily marital to parent–child spillover: individual and family systems approaches. Fam. Relat. 72, 1675–1693. doi: 10.1111/fare.12777

[ref19] Kuo P. X. Volling B. L. Gonzalez R. (2017). His, hers, or theirs? Coparenting after the birth of a second child. J. Fam. Psychol. 31, 710–720. doi: 10.1037/fam0000321, PMID: 28368201 PMC5608629

[ref20] le Y. McDaniel B. T. Leavitt C. E. Feinberg M. E. (2016). Longitudinal associations between relationship quality and coparenting across the transition to parenthood: a dyadic perspective. J. Fam. Psychol. 30, 918–926. doi: 10.1037/fam0000217, PMID: 27183188 PMC5112151

[ref21] Little R. J. A. (1988). A test of missing completely at random for multivariate data with missing values. J. Am. Stat. Assoc. 83, 1198–1202. doi: 10.1080/01621459.1988.10478722

[ref22] Maurer T. W. Pleck J. H. Rane T. R. (2001). Parental identity and reflected-appraisals: measurement and gender dynamics. J. Marriage Fam. 63, 309–321. doi: 10.1111/j.1741-3737.2001.00309.x

[ref9001] Maurer T. W. Pleck J. H. (2006). Fathers’ caregiving and breadwinning: A gender congruence analysis. Psychology of Men and Masculinity, 7, 101–112. doi: 10.1037/1524-9220.7.2.101

[ref9002] Muthén L. K. Muthén B. O. (1998–2022). Mplus User’s Guide. Los Angeles, CA: Muthén and Muthén.

[ref9004] Nguyen D. (2018). Fatherhood: Motivations for Paternal Involvement (Doctoral dissertation, Lehigh University). Available at: https://preserve.lehigh.edu/etd/4311

[ref24] Park B. Smith J. A. Correll J. (2010). The persistence of implicit behavioral associations for moms and dads. J. Exp. Soc. Psychol. 46, 809–815. doi: 10.1016/j.jesp.2010.04.009

[ref25] Peltz J. S. Rogge R. D. Sturge-Apple M. L. (2018). Transactions within the family: Coparenting mediates associations between parents’ relationship satisfaction and the parent–child relationship. J. Fam. Psychol. 32, 553–564. doi: 10.1037/fam0000413, PMID: 29927285

[ref26] Ronaghan D. Gaulke T. Theule J. (2024). The association between marital satisfaction and coparenting quality: a meta-analysis: journal of family psychology. J. Fam. Psychol. 38, 236–245. doi: 10.1037/fam0001149, PMID: 37747533

[ref27] Satorra A. Bentler P. M. (2010). Ensuring Positiveness of the scaled difference chi-square test statistic. Psychometrika 75, 243–248. doi: 10.1007/s11336-009-9135-y, PMID: 20640194 PMC2905175

[ref28] Schoppe-Sullivan S. J. Fagan J. (2020). The evolution of fathering research in the 21st century: persistent challenges, new directions. J. Marriage Fam. 82, 175–197. doi: 10.1111/jomf.12645

[ref29] Schumm W. R. Paff-Bergen L. A. Hatch R. C. Obiorah F. C. Copeland J. M. Meens L. D.. (1986). Concurrent and discriminant validity of the Kansas marital satisfaction scale. J. Marriage Fam. 48, 381–387. doi: 10.2307/352405

[ref30] Trivers R. L. (1972). “Parental investment and sexual selection” in Sexual selection and the descent of man (Chicago: Aldine-Atherton), 136–179.

[ref31] Volling B. L. Palkovitz R. (2021). Fathering: new perspectives, paradigms, and possibilities: psychology of men & masculinities. Psychol. Men Mascul 22, 427–432. doi: 10.1037/men0000354

